# Free-Weight Resistance Training in Youth Athletes: A Narrative Review

**DOI:** 10.1007/s40279-020-01307-7

**Published:** 2020-06-23

**Authors:** Stephen J. McQuilliam, David R. Clark, Robert M. Erskine, Thomas E. Brownlee

**Affiliations:** 1 School of Sport and Exercise Sciences, Liverpool John Moores University, Liverpool, UK; 2 Institute of Sport, Exercise and Health, University College London, London, UK

## Abstract

Generating high levels of muscular strength and power are important for success in sport and may have long-term implications for sporting careers in youth athletes. Importantly, maturation may confound the neuromuscular adaptations to resistance training when attempting to differentiate between training- vs. growth-induced strength and power gains; thus, potentially leading to erroneous conclusions regarding the efficacy of resistance training in youth athletes. The aim of this review was to critically appraise the literature concerning the efficacy of externally loaded free-weight resistance training on strength and power measures in youth athletes at different stages of maturity. Strength underpins power production; thus, developing strength through traditional resistance training methods can positively influence powerful sporting movements. In addition, weightlifting has the capacity to improve muscular power via explosive lower-body triple extension, which is essential for many sports. Despite the complexity of the techniques involved, it can be a safe and effective method to improve athletic qualities in young athletes, potentially more so than plyometric training. While low-load, high-velocity training can have a positive effect influence on high speed movements such as sprinting, the reduced intensity appears to be disadvantageous post peak-height velocity. Irrespective of age, well-coached progressive strength training adhering strictly to correct technique can then be periodised within a long-term athletic development program. It is important to primarily develop muscular strength, while concurrently refining the technical skill required for weightlifting. Physically mature athletes should undertake high-intensity resistance training to maximise neuromuscular adaptations, leading to positive changes in strength and power.

## Key Points

**Table:** 

Irrespective of age, following an initial focus on fundamental movement techniques, strength development can be periodised within a long-term athlete program.
As strength fundamentally underpins power, it is important to first develop this, while concurrently refining the technical skills required for weightlifting.
Technically able physically mature athletes should undertake high-intensity resistance training (≥ 80% 1RM) to maximise neuromuscular adaptations, leading to changes in strength and power.

## Introduction

In many sports, the ability to generate high levels of muscular power is an important component for success [1]. Thus, practitioners aim to improve muscular power via effective and efficient training programs. Previously, a potentially misleading report based on the hospital admission records of injury cases concluded that resistance training (RT) was not safe in youth populations [2]. Further investigation determined that many of the recorded injuries were accidents resulting from incorrect exercise technique and/or poor supervision. More recent research has shown that RT in youth athletes can be a safe and beneficial training method [3, 4]. Researchers have proposed that RT interventions in youth populations result in significant increases in strength, power and agility and reduced injury risk [5, 6]. However, a point of conjecture surrounds RT best practice and the translation of this from the literature to the applied setting.

For young athletes, development of strength and power may have long-term implications for their sporting careers. The confounding factor of maturation on training adaptations in terms of training- vs. growth-induced strength/power gains can complicate the training process. For example, chronologically older youth soccer players have been shown to be stronger in absolute terms but not when strength was normalised to body mass [7, 8]. Environmental factors, such as time restrictions due to sport-specific technical training and a limited number of appropriately qualified and experienced staff available to supervise and implement RT can also make translation of scientific literature into applied practice challenging [9].

The aim of this review was to critically analyse the scientific literature regarding the use and efficacy of RT regarding neuromuscular adaptations and how they translate into strength and power gains in youth sport. The objective is to provide recommendations based on the available evidence in the literature on the best practice regarding RT in youth sport, with particular reference to maturity status.

## Long-Term Athlete Development Models

Practitioners and researchers have attempted to align various training methods with each stage of maturity in young athletes. As such, several training models have been proposed. Long-term athlete development (LTAD) models may aid in structuring a young athlete’s training. An early well-known LTAD framework was proposed by Côté [10], outlining three clear phases: sampling years (ages 6–12 years), specialising years (ages 13–15 years) and investment years (ages 16 + years). However, a potential problem is the classification of athletes based on the chronological age [11]. The chronological age of an individual is defined as a selected time point from date of birth [12]. In youth sport, chronological age is typically utilised to categorise age groups for competitions/academy squads [13]. However, individuals of the same chronological age can differ greatly in terms of biological age [14], which is defined as the stage of an individual’s physical growth in relation to skeletal, sexual or somatic attributes [15]. The LTAD model by Bayli and Hamilton [16] attempted to correct for this by using biological age, through longitudinal monitoring of somatic variables. This allows practitioners to identify time periods of accelerated growth, regarding peak-height velocity (PHV, the phase where peak rate of skeletal growth occurs) and peak-weight velocity (PWV, the phase where peak rate of maturation-associated skeletal muscle accretion occurs) for a specific athlete and programme training accordingly [16]. The timing and speed of biological maturation is highly individual and, therefore, it is an important factor to consider when designing a training program, as it has been proposed to be more appropriate than chronological age [17].

The LTAD model suggested by Bayli and Hamilton [16] has been structured utilising supposed “windows of opportunity”, during which certain physiological characteristics are theoretically more responsive to training stimuli. However, this theory lacks evidence due to the diversity and range of components that contribute to a variety of sports [11]. In contrast, the Youth Physical Development (YPD) model suggests that most physical qualities are trainable throughout maturation, with different mechanisms underpinning adaptations [17]. A meta-analysis revealed that youth athletes may benefit from RT to the same extent, independently of age [6], although a later review suggested that absolute increases in strength were greater during or after PHV than those seen pre-PHV [18]. Owing to dissimilar reports, the results should be interpreted with caution. These models provide a guideline for effective training prescription; however, differences in training history, biological age and sporting requirements will influence the implementation and resulting adaptations.

## Influence of Maturation on Strength, Power and Speed

Maturity status has been identified as a contributing factor to a variety of different physical performance indicators, such as strength, peak power, sprinting, change of direction speed (COD), as well as both anaerobic and aerobic performance [19, 20]. It is important to track biological maturation longitudinally, as those who mature early generally have an advantage over their late-maturing peers due to greater strength and power at that point in time. In addition, there may be a disproportionate number of young athletes with birth dates in the first quarter of the selection year due to an advanced maturity status, known as the relative age effect [21]. Although individuals may be physically dominant during adolescence due to advanced maturation, this may not continue to be the case when fully mature [13]. During adolescence, it is possible to be at very different stages of biological maturation with the same chronological age, thus practitioners need to be aware that individuals within the same cohort may require different training stimuli.

The biological changes that occur from childhood through to full maturity directly influence strength and power via multiple mechanisms. Prior to PHV, increases in strength and power via training are suggested to be a result of improved neuromuscular activation [11, 19, 22, 23]. During this stage of maturation (pre-PHV), relatively low concentrations of circulating androgens, such as testosterone and growth hormone, limit the capacity for skeletal muscle morphological adaptations [24]. A significant phase of growth starts in girls and boys between 9–12 and 12–14 years old, respectively. In relation to biological maturation, this equates to approximately 1.5 years prior to PHV [11]. This period of elevated growth rate lasts until 0.5–1 year post-PHV [19], during which time another large increase in muscular power occurs [11]. This increase in strength occurs in both sexes, but more so in boys due to more testosterone being secreted by the testes than the ovaries [25]. As testosterone is a potent stimulator of muscle protein synthesis [26] and inhibitor of muscle protein breakdown [27], it follows that, during this period, boys more than girls experience a significant accretion of muscle mass, which is the main physiological determinant of maximum strength [28]. Muscular strength is the ability to apply force on an external object. As the ability to generate force improves, more force can be applied in the same time frame, resulting in greater power production. As such, similar physiological mechanisms may drive maturation-associated increases in both attributes [29]. During adolescence, males exhibit a re-distribution of muscle fibre type from predominantly type I to type II fibres [30, 31]. Type II fibres have a greater cross-sectional area (CSA), allowing for greater force production than type I fibres as well as a faster cross-bridge attachment/de-attachment cycle allowing for a faster shortening velocity, thus greater power production [32]. In addition, an increase in limb length during skeletal growth may simultaneously increase the internal moment arm, thus increasing torque production [23].

Although there are large increases in strength and power during PHV, the greatest gains tend to occur at onset of PWV. This is typically between 6 months to a year after PHV, when the rate of lean mass accruement is greatest [33]. In relation to muscle morphology, three key factors influence power generation: muscle physiological CSA, muscle fascicle length and muscle fascicle pennation angle [34], all of which can be assessed non-invasively via ultrasonography. Briefly, muscle physiological CSA represents the CSA of the total number of muscle fibres, perpendicular to their long axis [35]. Muscle fascicle length is defined by the number of sarcomeres in series, with longer muscle fibres able to contract faster than shorter fibres. Muscle fascicle pennation angle, i.e. the angle at which fascicles insert into the aponeurosis, increases due to fibre hypertrophy, which is caused by an increase in the number of sarcomeres arranged in parallel [34]. Muscles with larger fibre CSAs and greater pennation angles produce greater forces, while muscles with longer fascicles and smaller pennation angles have a greater shortening velocity [34, 36]. Interestingly, muscle fascicle length and pennation angle appear to be independent parameters of maturation. There appears to be no difference in muscle fascicle length when normalised to body height and muscle fascicle pennation angle seems to be comparable between children, adolescents and adults [37–39]. However, it should be noted that 2D ultrasound imaging of muscle architecture may not accurately quantify differences in a 3D structure, particularly if extrapolation of fascicle measurements is necessary due to limitations with transducer width [40]. Further, there are limited data regarding natural development of the aforementioned physical attributes in terms of longitudinal studies and the impact physical training may have.

To contextualise the above points, increases in strength in the absence of gains in body mass have a greater impact on sports where athletes propel their own body mass, for example sprinting and jumping. Alternatively, increases in strength with gains in body mass which are seen during PWV, may have greater influences where both high-force movements and momentum become important in sports such as rugby when tackling and breaking tackles. Realistically, practitioners should expect increases in absolute strength as a consequence of lean accretion with maturation, while increases in strength normalised to body mass are more likely the product of specific exercise training [7].

### Response to Training

There are no minimum age guidelines for youth participation in RT. National governing bodies for strength and conditioning support RT for children when the child is both physically and mentally prepared to engage in sport [3, 41]. This is determined based on their ability to follow instruction, which is central in ensuring safety [41].

As previously mentioned, adaptations differ according to maturity status in youth cohorts [11]. Load–velocity profiles can estimate maximal force, peak power and velocity capabilities in the assessed movement. Meylan et al. [42] reported different force–velocity–power (kinetic) responses between biological age groups. Following an 8-week RT intervention pre- and mid-PHV cohorts experienced increases in maximal velocity, which in turn improved maximum power on a machine-squat, whereas the post-PHV group expressed increases in power via improved maximal force and velocity. Benefits were more pronounced in the post-PHV group, particularly where high levels of force and power were required, such as 1RM strength test and 10 m acceleration. Furthermore, Rodríguez-Rosell et al. [43] implemented a low-load, high-velocity RT intervention, applying the same duration and frequency in youth soccer players. All groups (U13, U15 and U17) showed significant improvements in strength, jump and sprint assessments, although the degree of improvement diminished with increasing chronological age. The authors concluded that mature athletes require greater relative training loads to maximise adaptations, based on the higher relative maximal strength or 1RM [43].

The training status of those included in interventions and subsequent reviews is an important factor as this may influence efficacy of an intervention. In a systematic review and meta-analysis by Behm et al. [44], data indicated that untrained youth experience larger increases in both jump and sprint assessments than their trained counterparts due to inferior baseline results and RT being an unfamiliar stimulus. Behm et al. [44] proposed that trained youth might adapt through neural and morphological adaptations whereas untrained participants will improve primarily via neural pathways. Untrained youth may encounter a greater learning effect due to their relative inexperience. Therefore, untrained subjects may have to initially improve their motor-unit recruitment before morphological changes can be observed [45]. However, a subsequent review by Slimani et al. [46] reported no significant effect of training status on improvements in squat jump (SJ) performance. Slimani et al. [46] attributed the variances in findings to differences in methodology, as they focused on vertical jump performance exclusively, whereas Behm et al. [44] included other parameters, such as strength and sprint speed. When considering this potential greater trainability, methods derived from research in untrained youth populations should be implemented in high-level (trained) youth athlete settings with caution.

Despite a plethora of research examining outcome measures i.e. vertical jump and sprint performance, the training history of the participants involved is key. In addition, there appears to be limited research on the underpinning mechanisms behind these physiological adaptations to RT in adolescent athletes [47]; thus further investigation is required.

## Resistance Training Methods

The term “resistance training” is an all-encompassing term used throughout the literature referring to a variety of methods, primarily machines and/or free-weights. These methods have the capacity to augment both muscle physiological CSA and neural activation, which influence muscle strength and power. This section will focus on interventions primarily utilising free-weight RT as well as the commonly used Smith machine. Although the Smith machine it is not a free-weight exercise, it has a prominent place in strength-training research [48]. A number of free-weight training methods can induce positive adaptations in strength and power in youth cohorts, including heavy strength training, weightlifting (WL), peak-power training and a combination of these [49–51]. However, different modalities appear to be more beneficial depending on physical characteristics targeted and stage of maturity. Free-weight training refers to a load that moves freely in space, e.g. the barbell back squat and that is not attached to another support structure. Free-weight RT is seen as a more efficient method of improving strength. This allows for large compound movements coupled with reduced stability; therefore, increasing the recruitment of stabilising musculature around the primary muscles as well as superior reproduction of sporting actions, such as vertical jumping [6]. The following sections will aim to examine various free-weight RT modalities in youth populations and the influence on strength and power.

### Strength Training

Maximal strength underpins athletic muscular performance qualities, such as peak power, by increasing maximal force potential [52]. A significant correlation exists between higher relative training intensities (%RM) and improvements in maximal strength and motor skill performance in youth populations [45]. High levels of strength may influence sport-specific skills and increase jump height and sprint performance [53, 54]. Strength training can be defined as high-load RT relative to an individual’s maximal strength (≥ 80% 1RM), utilising two to four sets at low-repetition ranges (≤ 6) [55]. Furthermore, a recent meta-analysis highlighted that the most effective intensity to improve strength in youth athletes is 80–89% 1RM [6]. Training at high percentages of maximal strength has an important role in changing tendon properties in adolescent athletes [37]. Moreover, reduced tendon strain via an RT-induced increase in tendon CSA may reduce occurrence of tendinopathies [56]. High-intensity RT appears to be a fundamental component of a training regime in order to prepare a young athlete for sports participation via increased proxies of performance and reduced risk of injury.

As mentioned previously, improving an athlete’s strength may increase both initial acceleration and maximal sprint speed. Improving initial acceleration may be highly beneficial in sports such as soccer with approximately 60 accelerations occurring per match [57]. Impulse (the product of force multiplied by time) is a key determinant of acceleration. However, as time is restricted during the ground contact phase, maximising force production within this time window is vital [58]. There is a strong correlation between absolute squat strength and sprint performance due to an associated greater rate of force development (RFD) and in turn, ground reaction force [59, 60]. Thus, maximal strength has been identified as an important factor to maximise initial acceleration, where ground contact times > 200 ms enable greater force transfer [59]. These measures require the recruitment of the lower body musculature in one coordinated movement; consequently, the squat has become the cornerstone of many strength-training programs [51, 61].

When programmed appropriately, adolescents respond positively to high-intensity RT (> 80% 1RM). Keiner et al. [62] compared front and back squat strength in adolescent soccer players and weightlifters. As expected, weightlifters outperformed soccer players at all age groups. Notably, in the 17–19 years age group, youth weightlifters demonstrated 2.1 ± 0.1 × bodyweight 5RM back squat in comparison to the soccer players who produced a 1.3 ± 0.2 × bodyweight 4RM. Importantly, the weightlifting group completed the test to full-depth and for an extra repetition, whereas the soccer players were limited to parallel depth (thighs parallel to the floor). The full-squat results in a lower load as compared to the parallel squat, meaning that differences in strength between the two cohorts may be greater than reported [63]. These studies highlight the efficacy of high-intensity RT in youth populations.

#### Volume and Intensity

Training programs consist of numerous variables, more than just exercise selection. When designing a RT program two key components are primarily considered: training volume and training intensity. There are multiple ways to calculate both components. Volume may be quantified via a repetition, volume load and volume index method and intensity as percentage of 1RM or average load for an exercise/overall session, for instance [64]. In a sport setting the demands of competition and the time available may influence these variables, meaning time efficient training methods are of great value. When working with youth athletes, biological maturity and phases of accelerated growth must also be considered as part of LTAD [65].

#### Intensity

When the training aim is to build strength in youth athletes high-intensity RT (> 80% 1RM) has been suggested to be the most effective method and benefits can become apparent within a small timeframe [6]. Chelly et al. [51] implemented an 8-week, twice-weekly squat training program, comprising three sets at intensities between 80 and 90% 1RM in U18 soccer players with no RT experience. These loads were chosen because they are suggested to increase RFD, particularly in weaker/untrained participants [29, 52]. Along with a low-RT volume, there were significant increases in peak power, 40 m sprint, SJ and repeat bounding performance with no increases in thigh CSA; thus the adaptations were suggested to be neurological. Despite improvements in SJ, there were no significant improvements in countermovement jump (CMJ). Speirs et al. [66] reported similar results after a 5-week intervention, utilising 75–92% 1RM in U19 rugby union players with at least one year of RT experience. Although shorter in duration and a similar weekly RT volume to Chelly et al. [51], there were significant increases in back squat 1RM, 40 m sprint time and agility performance. In combination, there is evidence to suggest high-intensity RT with low-training volume can have an impact on acceleration, jump and COD in a short time period (Table 1), especially if the stimulus is unfamiliar.

**Table 1:** Results of commonly used performance measures from referenced high-intensity strength training studies

Study	Sport	Training type	Age (year)/squad	Volume (sets/reps/intensity)	Total weeks/sessions per week		1RM strength back squat (kg)	Squat jump height (cm)	CMJ height (cm)	10 m sprint (s)	20 m sprint (s)
Chelly et al. [51]	Soccer	RT: Back squat	RT: 17 ± 0.5	3/2–4	8/2	Pre	105 ± 14	31.5 ± 4	33.8 ± 4		
				80–90% 1RM		Post	142 ± 15*	34.6 ± 3*	36.3 ± 3		
Hammami et al. [67]	Soccer	RT: Back squat	RT: 16.2 ± 0.6	4–7/3–8	8/2	Pre	99.8 ± 7.5	36 ± 3	37 ± 5	1.92 ± 0.09	3.24 ± 0.03
				70–90% 1RM		Post	125.1 ± 4.7*	43 ± 2*	42 ± 4*	1.73 ± 0.01*	3.06 ± 0.02*
Sander et al. [70]	Soccer	RT: Back + front squat	Under 17 s	5/4–10	2 years/2	Pre	61.2 ± 10			1.746 ± 0.042	3.020 ± 0.067
				4–10 RM		Post	120.4 ± 11.4*			1.712 ± 0.045*	2.961 ± 0.058*
			Under 15 s			Pre	52 ± 10.7			1.802 ± 0.082	3.120 ± 0.140
						Post	113 ± 15.2*			1.731 ± 0.078*	2.984 ± 0.126*
			Under 13 s			Pre	25 ± 9.6			1.917 ± 0.056	3.375 ± 0.101
						Post	90 ± 13.5*			1.813 ± 0.078*	3.194 ± 0.142*
Styles et al. [68]	Soccer	RT: Back squat + Romanian deadlift	18.3 ± 1.2	3–4/3–5	6/2	Pre	125.4 ± 13.78			1.83 ± 0.05	3.09 ± 0.07
				90% 1RM		Post	149.3 ± 16.62*			1.78 ± 0.05*	3.05 ± 0.05*

*RT* resistance training, *RM* repetition maximum, *CMJ* countermovement jump

*Significant difference

#### Volume

Alongside training intensity, the volume of training must be considered in the athletic development of youth athletes. Both Hammami et al. [67] and Styles et al. [68] implemented twice-weekly high-intensity RT interventions in adolescent athletes over 8 and 6 weeks respectively. Both studies reported significant improvements in strength and 20 m sprint time. The largest improvements in the study by Hammami et al. [67] occurred in 5 m (11.1%) and 10 m (9.4%) sprint tests, which is in line with the previous research [54]. Furthermore, the largest improvements in agility were observed in tests that required a greater number of direction changes. The greater number of accelerations and decelerations associated with multiple direction changes allow for more instances, where greater strength could influence the test outcome. Hammami et al. [67] also reported significant increases in both SJ and CMJ. This is in contrast to the previous research where only increases in SJ were seen [51]. Although both interventions incorporated high-intensity loads between 70 and 90% 1RM, the main difference in protocol was training volume. Whereas Chelly et al. [51] implemented eight sets per week totalling 18 repetitions, Hammami et al. [67] completed 42 sets, totalling 186 repetitions. Training volume has been highlighted as an important stimulus for adaptation in athletic populations [69]. However, despite a far lower training volume than Hammami et al. [67], Chelly et al. [51] also produced significant increases in 1RM half-squat strength, squat jump, as well as 5 m and 40 m sprint performance in the same population. Irrespective of volume, both Chelly et al. [51] and Hammami et al. [67] attributed the improvements to neural adaptations, with neither study finding significant changes in thigh CSA, which is in line with a previous review by Ford et al. [11]. A multitude of factors can influence adaptation including previous RT experience, biological age as well as training volume and intensity [6, 18, 44] but it appears that higher training intensities (> 80% 1RM) and low volume can lead to similar increases in performance as low-intensity, high volume (Table 1).

#### Long-Term Training

The previously mentioned studies were all short in duration (e.g. ≤ 8 weeks) in chronologically older youth athletes, where biological growth might not be significant enough to affect the RT-induced changes in strength or power. Sander et al. [70] conducted a 2-year intervention in young soccer players to observe the influence of regular RT alongside soccer training on strength and sprint performance. At the start of the intervention, groups consisted of players from U13, U15 and U17 squads matched with control groups performing only soccer training. Key low-body exercises included both the front and back squat, as well as the deadlift, all at an intensity of 75 to 90% 1RM. The largest effect size was seen in the U13s for both squat tests (back squat ES = 2.0, front squat ES = 1.9). This is supported by Lesinski et al. [6], where larger effect sizes were seen in younger (≤ 13 years) than adolescent males (14–18 years) (ES = 1.35 vs. 0.91). Strength training also significantly improved 30 m sprint performance at all 5 m intervals when compared with the control group in both the U13s and U15s squads. The U17s failed to improve 10 m sprint performance, which may have been due to more variability in the percentage change [70]. Of the control groups, the U13s and U15s improved 15–30 m and 10–30 m sprint interval performance, thus suggesting maturation and/or soccer training influenced these variables. However, the U17s control group did not improve any sprint times. Because this group would not be expected to demonstrate significant maturation-related growth, these results suggest that soccer-specific training does not improve sprint performance and that the improvements in the U13 and U15 control groups were due to maturation-related growth, not soccer-specific training [70]. However, maturity status was not assessed and therefore could not be used a covariate in subsequent analysis to delineate the RT effect from the soccer training effect. Nonetheless, Sander et al. [70] highlights that long-term RT in adolescent athletes is effective in improving strength and in turn sprint performance in youth athletes from 5 to 30 m.

When youth athletes are systematically exposed to high-intensity RT over a prolonged period of time (e.g. ≥ 12 months) there can be significant increases in maximum strength as well as sport-specific assessments, such as vertical jumps and sprinting performance (Table 1). The literature suggests that benefits can be seen at all stages of biological maturity; therefore, high-intensity RT should be included throughout an athlete’s development.

### Weightlifting

As previously stated, high levels of muscular power are important for sports performance [1]. Training to increase maximum strength augments the capacity to develop power [71]. A holistic training program that incorporates maximum strength and WL variations can facilitate this transfer [72]. In WL movements, the emphasis is typically on movement speed, at moderate to heavy loads. As a result, WL can increase motor-unit recruitment and therefore RFD [73]. The two primary WL lifts are the clean and jerk and the snatch, with derivatives, such as the hang-power clean involving high force and velocity outputs [72], which are the components of power. Mechanically, WL movements align with the principle of specificity by replicating kinematic and kinetic characteristics of the vertical jump [74]. In contrast to traditional RT methods, there is limited research on WL in youth populations.

Despite limited research in the area, results appear to be promising at each stage of biological maturity. Chaouachi et al. [50] compared WL to traditional RT and plyometric training (PLYO) in 10- to 12 year-old males for a period of 12 weeks. The RT intervention utilised squats and lunges, whereas the WL program implemented clean and snatch variants. Both groups followed identical set and repetition schemes in an attempt to equalise training volume (i.e. 1–3 sets × 8–12 reps). The results showed no clear differences between RT and WL in terms of increases in 5 m acceleration, 20 m flying sprint or vertical CMJ performance but there was a likely benefit for WL in horizontal CMJ distance. Importantly, there were larger effect sizes for the WL group when compared with the PLYO group for changes in all strength and power variables. Subjects had no previous RT experience and the concentric phase of the squat was not explosive which should be considered when interpreting these results. In an adolescent cohort with limited RT experience, Channell, Barfield [75] compared the effect of WL *vs.* traditional RT on vertical CMJ performance. Each intervention group shared a number of common exercises, while completing three group-specific core lifts. Similar to Chaouachi et al. [50], after 8-week, both groups saw similar improvements in vertical CMJ performance. Taken together, the results suggest that WL may be more effective when improving muscular power and subsequent athletic tasks than PLYO in young populations.

Following a period of WL, Channell and Barfield [75] suggested improvements in strength and power seen were likely due to neural adaptations, i.e. greater neuromuscular voluntary activation of the agonists, synergists and stabilisers, all contributing to improve technique, as well as muscular force and contraction velocity. Arabatzi and Kellis [76] examined differences in EMG activity between WL and traditional RT to explain why WL may produce better jump performance. They implemented an 8-week high-intensity (80–90% 1RM) intervention in resistance-trained male students, comparing WL variants to traditional RT. They concluded that greater improvements in SJ, CMJ and drop jump (DJ) with WL were due to increased agonist muscle (rectus femoris) activation, reduced antagonist muscle (biceps femoris) co-activation and an increased leg stiffness. The RT group also showed an increased leg stiffness, seen as a decreased change in the body’s centre of mass during the eccentric phase of a drop jump test. However, there was an increased activation of both agonist and antagonist muscle groups. In powerful actions, such as jumping, increased antagonist muscle co-activation may reduce velocity towards the end of the movement, limiting power production [77]. The results of Arabatzi and Kellis [76] highlight that ballistic RT in the form of WL may produce a more beneficial neural activation pattern of agonist and antagonist muscles that is not prevalent with traditional RT. Thus, incorporating WL into a training program appears to be important for improving ballistic actions.

Weightlifting is a training method that has the capacity to improve muscular power by utilising the explosive lower body triple extension, which is essential for sprinting and jump variants in many sports [1]. Despite the complexity of WL, it can be an effective method to improve athletic qualities in young athletes at each stage of biological maturity (Table 2), with minimal injury risk when appropriately supervised [50].

**Table 2:** Results of commonly used performance measures from referenced weightlifting training studies

Study	Sport	Training type	Age (year) /squad	Volume (sets/reps/intensity)	Total weeks/sessions per week		1RM strength (kg)	Squat jump height (cm)	CMJ height (cm)	10 m sprint (s)	20 m sprint (s)
Chaouachi et al. [50]	Judo and wrestling	WL	11.1 ± 1	1–3/8–12	12/2	Effect Size			Large		
		RT	11.1 ± 1	Not specified		Effect Size			Small		
Channell and Barfield [75]	American football	RT	15.9 ± 1.2	3–5/3–10	8/3	Pre	132.6 ± 30.94		47.2 ± 9.5		
				75–95% 1RM		Post	128.3 ± 26.01		48.3 ± 8.9		
		WL				Pre	144 ± 41.6		57.5 ± 7.2		
						Post	161.6 ± 29.3		60.1 ± 3.9		

*WL* weightlifting, *RT* resistance training, *CMJ* countermovement jump, *RM* repetition maximum

*Significant difference

### Peak-Power Training

Training methods focusing on low-load and high-velocity movements are suggested to be beneficial for sprinting as well as vertical and horizontal jump performance, particularly in pre-PHV athletes [78]. Much like WL, training of this nature centres on peak-power production, which can occur at different intensities according to the exercise. Cormie et al. [79] reported peak power with external loads occurred at 0%, 54% and 80% of 1RM jump squat, back squat and power clean, respectively. However, always training at peak power may limit further improvements, as strength would remain underdeveloped [29]. This is important, as greater levels of strength relative to body mass correlate strongly to CMJ height [80].

This method has been suggested to improve strength and power via neural mechanisms, making it ideal for pre-PHV athletes, when morphological adaptations are limited. However, post-PHV, increases in peak power is predominately via an increase in force generation [42]. Thus, training at peak power would result in a sub-optimal training load. In a long-term study, Gonzalez-Badillo et al. [81] implemented a twice-weekly, high-velocity RT intervention in Spanish academy soccer players, where they utilised low volumes of squats at loads of ~ 45–59% 1RM combined with jump and sprint training. Interestingly, after 6 months of RT, both the U16 and U18 groups matched or outperformed the U21 control group in the iso-inertial squat strength test, vertical CMJ and 20 m sprint performance tests. Thus, it could be argued that 6 months of RT produced similar or greater gains than 5 years of soccer training and maturation combined. However, a limiting factor is that there were no measures of maturity or age-matched control groups, as maturation will have likely influenced these results. Rodríguez-Rosell et al. [43] employed a similar design but incorporated age matched controls, together with U13, U15 and U17 male soccer players who had no prior RT experience. However, the intervention was much shorter in duration. Over 6 weeks, a combination of high-velocity full squats (45–60% 1RM), jumps, sprints and COD drills significantly improved iso-inertial squat load at 1.00 m·s^−1^ (~ 56% 1RM), vertical CMJ and 20 m sprint performance in all groups, with the percentage increase reducing with advancing chronological age. Whereas the U13s and U15s were significantly better than their age-matched control group in all measures post training, the U17s outperformed their control group in the sprint and strength assessments only (Table 3). The results presented here can be explained by the different kinetic responses that increase peak power at different stages of biological maturity [42]. While pre- and mid-PHV individuals appear to increase peak power primarily via increasing movement velocity, post-PHV individuals appear to do this principally by increasing force output [42]. Although there were increases in strength in the U17 age group the different kinetic responses reported by Meylan et al. [42] suggest that low-load, high-velocity training is sub-optimal at the later stages of biological age. In addition, benefits from this method are potentially due to limited/no-previous RT experience; thus, it is less likely to have an effect with increased training age. This suggests that low-load, high-velocity training may be more beneficial for younger than for more mature athletes. For physically mature athletes, however, the incorporation of high-intensity strength training is likely required to elicit greater improvements in performance.

**Table 3:** Results of commonly used performance measures from referenced high-velocity, low-load training studies

Study	Sport	Training type	Age (year)/squad	Volume (sets/reps/intensity)	Total weeks/sessions per week		1RM strength (kg)	Squat jump height (cm)	CMJ height (cm)	10 m sprint (s)	20 m sprint (s)
Gonzalez-Badillo et al. [81]	Soccer	Back squats + loaded CMJ	U16: 14.9 ± 0.3	2–4/6–8	26/2	Pre			35.4 ± 3.9		2.99 ± 0.10
				~ 45–60% 1RM		Post			39.1 ± 4.9*		2.97 ± 0.09
			U18: 17.8 ± 0.4			Pre			38.4 ± 3.0		2.96 ± 0.10
						Post			41.3 ± 4.5*		2.92 ± 0.10*
Rodríguez-Rosell et al. [43]	Soccer	Back Squat	U13: 12.6 ± 0.5	2–3/8–4	6 / 2	Pre	38.6 ± 17.9		26.6 ± 4.3	1.9 ± 0.06	3.38 ± 0.12
				~ 45–60% 1RM		Post	57.2 ± 15.9*		29.8 ± 3.9*	1.84 ± 0.07*	3.29 ± 0.12*
			U15: 14.6 ± 0.5			Pre	64.0 ± 14.5		32.4 ± 5.2	1.78 ± 0.06	3.13 ± 0.11
						Post	81.7 ± 16.6*		35.7 ± 6.1*	1.75 ± 0.06*	3.09 ± 0.11*
			U17: 16.5 ± 0.5			Pre	91.2 ± 12.9		37.8 ± 5.1	1.72 ± 0.06	2.99 ± 0.40
						Post	103.5 ± 17.3*		40.0 ± 5.6*	1.68 ± 0.06*	2.95 ± 0.09*

*CMJ* countermovement jump, *RM* repetition maximum

*Significant difference

### Combined Methods Resistance Training

As part of a holistic RT program, it is unrealistic to implement one method in isolation as seen in certain studies [50, 51, 81]. Aspects of the force–velocity curve are involved in many sporting actions. The synergistic benefits of a combined-method approach on improvements in sprint performance have previously been acknowledged. In a systematic review and meta-analysis, the largest effect sizes on sprint performance were seen when back squat, loaded SJ/CMJ and PLYO were implemented concurrently (ES = − 1.20) [60]. The effect was much greater as compared to back squat (ES = − 0.81) and loaded jump training alone (ES = − 0.29). A similar pattern for training intensity became apparent with a combination of high (> 85% of 1RM) and very light (< 40% 1RM) loads producing the largest effect size (ES = − 0.82). However, lower effect sizes where seen when high (ES = − 0.52) or low (40–59.9% 1RM, ES = − 0.16) loads were used in isolation. Interestingly, medium loads (60–84.9% 1RM, ES = − 0.97) in isolation produced the largest ES. With regards to volume, there was a moderate relationship with greater training frequency and sprint performance (*r* = 0.50; *p* = 0.001) and longer rest intervals (*r* = − 0.47; *p* ≤ 0.001) but no correlations for the number of exercises, sets or repetitions per set. Seitz et al. [60] suggested that a combination of high-, medium- and very light loads was the most effective approach to improve sprint performance. Although the participants in the review by Seitz et al. [60] (13–25 years of age) did not include those pre-PHV and continue into adulthood, it is important to note that Seitz et al. [60] reported a non-significant correlation for both age and height regarding the effect of RT on sprint performance. This demonstrates that a variety of external loads and, therefore, velocities are beneficial at all age groups.

Recently, the combination of both high- and low-load RT within a single training session has gained popularity due to its time efficiency, often referred to as complex training (CT) [82]. A systematic review found CT to be significantly more effective at improving 20 m sprint times and 1RM strength as compared to other RT methods, but acknowledged that single study outliers may have influenced this [83]. Although no significant differences were reported concerning changes in jump, 5 m, 10 m, 30 m and 40 m sprint performance between CT and other methods, the improvements were associated with lower training volumes. In addition, the authors proposed that the potentially novel exposure to high-intensity RT might have been responsible improvements following CT, as exposure to high-velocity movements would occur as a result of sport-specific training. Therefore, youth athletes may benefit from training at a range of intensities as part of their LTAD.

In youth cohorts, it is necessary to consider the influence of maturity status on the effectiveness of CT due to the different mechanisms responsible for training adaptations at each stage of maturity [11]. Both Lloyd et al. [49] and Radnor et al. [84] examined the adaptation to traditional RT, PLYO and CT in untrained pre- and post-PHV groups. In both short-term interventions, PLYO was a more effective training method to improve jump and sprint assessments in the pre-PHV groups, while CT was more effective at post-PHV [49, 84]. Radnor et al. [84] continued to specify that CT and RT were more effective for improving variables that required high concentric force production, such as initial acceleration, whereas PLYO was more effective at improving peak running velocity and reactive strength index [84]. Although the RT protocol differs from the suggestions of Lesinski et al. [6], the results support the incorporation of RT, particularly post-PHV, where greater increases in maximal force and thus peak power are possible [42].

To the authors' knowledge, only one long-term study has examined WL as part of a CT program in youth athletes. Pichardo et al. [85] examined the effect of incorporating WL into a RT program on isometric mid-thigh pull (IMTP), vertical CMJ, horizontal CMJ and 30 m sprint performance. The original RT program (CRT) already comprised traditional RT and plyometric movements (CRT). In the WLRT group, conventional exercises, such as the deadlift and a plyometric movement were replaced with WL exercises for 28 weeks (WLRT), split into initial light-load technique training followed by 14 weeks at higher training intensities. Both groups significantly improved IMTP, vertical CMJ, horizontal CMJ and sprint performance (10–30 m) to a similar extent post training. However, the timing of these changes varied between groups, with both groups increasing absolute IMTP force mid-way through the training period, but only the CRT group displayed improvements in both 20 m and 30 m sprint tests. From mid-way, training intensity increased in both groups and from mid- to post-training, there were greater percentage improvements in all jump and sprint measures as compared to pre- to mid-training, particularly in the WLRT group. The authors proposed that the increased intensity aided the transfer of improved strength into improved power. The study was not without its limitations, namely the absence of a control group and the inability to distinguish between the effects of training *vs.* biological maturation due to no measure of maturity. However, with this study, Pichardo et al. [85] demonstrated the importance of developing strength prior to power, and that longitudinal studies are required to demonstrate the translation of strength into power.

The results from Lloyd et al. [49], Radnor et al. [84] and Pichardo et al. [85] highlight the importance of incorporating both high-force and high-velocity training to improve a range of strength and power measures (Table 4). Improving maximum force production becomes increasingly important post-PHV, due to the maturation-associated muscle growth allowing greater force outputs to improve peak power [11, 42]. When incorporated alongside loaded ballistic exercises, research would suggest there are greater improvements in powerful dynamic sporting actions post training, providing strength has already been developed.

**Table 4:** Results of commonly used performance measures from referenced complex training studies

Study	Sport	Training type	Age (year)/squad	Volume (sets/reps/intensity)	Total weeks/sessions per week		Squat jump height (cm)	CMJ height (cm)	10 m sprint (s)	20 m sprint (s)
Lloyd et al. [49]	High-school physical education	Pre-PHV RT	12.6 ± 0.3	RT: 3/10	6/2	Pre	22.3 ± 4.9		2.3 ± 0.2	3.4 ± 0.3
			PHV: − 1.4 ± 0.6	75% 1RM		Post	24.8 ± 4.6*		2.2 ± 0.2*	3.4 ± 0.3
		Pre-PHV CT	12.7 ± 0.3			Pre	24.1 ± 4.3		2.2 ± 0.2	3.4 ± 0.3
			PHV: − 1.5 ± 0.7	CT: 2–5/3–10		Post	28.2 ± 4.6*		2.1 ± 0.2*	3.3 ± 0.3*
		Post-PHV RT	16.3 ± 0.3			Pre	32.4 ± 5.0		1.9 ± 0.1	2.8 ± 0.2
			PHV: 1.3 ± 0.3			Post	34.6 ± 5.1*		1.8 ± 0.1*	2.7 ± 0.2
		Post-PHV CT	16.2 ± 0.3			Pre	33.2 ± 5.5		1.9 ± 01	2.8 ± 0.2
			PHV: 1.3 ± 0.6			Post	37.4 ± 5.4*		1.8 ± 0.1*	2.6 ± 0.2*
Radnor et al. [84]	High-school physical education	Pre-PHV RT	12.6 ± 0.3	RT: 3/10	6 / 2	Change %	+ 16.56 ± 11.70%*		+ 3.1 ± 2.3%*	
			PHV: − 1.4 ± 0.6	75% 1RM						
		Pre-PHV CT	12.7 ± 0.3			Change %	17.7 ± 5.42%*		+ 3.34 ± 1.83%*	
			PHV: − 1.5 ± 0.7	CT: 2–5/3–10						
		Post-PHV RT	16.3 ± 0.3	75% 1RM		Change %	1.41 ± 2.15%*		0.37 ± 0.43%	
			PHV: 1.3 ± 0.3							
		Post-PHV CT	16.2 ± 0.3			Change %	12.93 ± 3.89%*		2.68 ± 1.10%*	
			PHV: 1.3 ± 0.6							
Pichardo et al. [85]	High-school performance program	WLRT	13.9 ± 0.6	1–5/2–12	28/2	Change %		+ 17.05 ± 23.41%*	− 24.79 ± 3.36%*	− 23.98 ± 2.87%*
			MO: 0.1 ± 0.9	Not stated						
		RT	14.0 ± 0.5			Change %		+ 9.14 ± 19.64	− 26.07 ± 10.96%*	− 25.30 ± 3.64%*
			MO: 0.3 ± 0.6							

*RT* resistance training, *CT* complex training, *WLRT* weightlifting and resistance training, *PHV* peak-height velocity, *CMJ* countermovement jump, *IMTP* isometric mid-thigh pull, *RMc* repetition maximum

*Significant difference

## Conclusion

The aim of this review was to critically appraise the scientific literature regarding the use and efficacy of RT in youth sport. Resistance training is an important aspect of a young athlete’s development and is a safe and effective method when appropriately planned and supervised [3]. Based on the available evidence in the literature, the objective of this review was to provide recommendations on best practice regarding intensity and volume of RT/WL in youth athletes with specific reference to maturity status, which we have highlighted during the review and summarised below.

### Practical Implications and Future Research

**Fig. 1:**
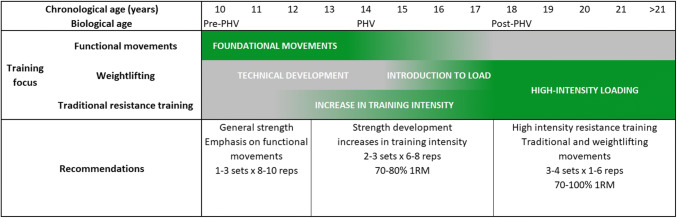
Evidenced-based recommendations for developing strength and power within a holistic long-term athletic development plan in youth athletes. Grey refers a lower focus, green refers to a greater training focus. *PHV* Peak-height velocity, *reps* repetitions, *RM* repetition maximum

Irrespective of age, following an initial focus on fundamental movement techniques, such as the squat and hip-hinge, strength development can then be periodised within a LTAD program. As strength fundamentally underpins power, it is important to first develop this, while concurrently refining the technical skill required for WL. Physically mature athletes should undertake high-intensity RT to maximise neuromuscular adaptations to RT, leading to changes in physical performance (Fig. 1).

It is important to consider that RT is a component of a larger sport-specific training structure, where time availability for RT/WL may be limited. Thus, future research should investigate various low-volume, high-intensity RT/WL training methods to determine the required volume of training to elicit improvements in physical performance and physiological adaptations in youth athletes.

## Data Availability

Data sharing was not applicable to this article as no datasets were generated or analysed during the current study.
